# The influence of quality of work life on motivation and retention of local government tuberculosis control programme supervisors in South-eastern Nigeria

**DOI:** 10.1371/journal.pone.0220292

**Published:** 2019-07-24

**Authors:** Daniel Chukwuemeka Ogbuabor, Ijeoma Lewechi Okoronkwo

**Affiliations:** 1 Department of Health Systems and Policy, Sustainable Impact Resource Agency, Enugu, Enugu State, Nigeria; 2 Department of Health Administration and Management, University of Nigeria Enugu Campus, Enugu, Enugu State, Nigeria; University of Toronto, Rotman School, CANADA

## Abstract

**Introduction:**

Significant gap exists in knowledge about employee-centred human resources practices that address motivation and retention of local government tuberculosis control programme supervisors (LGTBS) in Nigeria. The study examined the role of quality of worklife (QWL) in motivating and retaining LGTBS.

**Materials and methods:**

The study was conducted in south-eastern region of Nigeria comprising five states and 95 local government areas. The design was mixed-methods. We used cross-sectional survey to collect quantitative data on socio-demographic factors, QWL, motivation and retention from a total sample of LGTBS. The qualitative component involved focus group discussions (n = 3) with 26 LGTBS. Quantitative data were analysed using exploratory factor analysis, descriptive statistics, Spearman correlation, Mann-Whitney test, Kruskal-Wallis test and multiple linear regression. Qualitative data were analysed using a thematic framework approach.

**Results:**

The final 40-item QWL scale was found to be valid and reliable. The LGTBS had high QWL (M = 5.15, SD = 0.88) and motivation (M = 5.92, SD = 1.08), but low intention to leave their jobs (M = 2.68, SD = 1.59). Education significantly predicted satisfaction with overall QWL, work-family balance and work design; but tenure predicted satisfaction with work context. Work design and work-family balance significantly predicted motivation of LGTBS. Motivation mediated the relationship between QWL and intention to leave and accounted for 29% variance in intention to leave. Whereas LGTBS were motivated by responsibility, learning opportunities, achievement and recognition; they were dissatisfied with lack of flexible work schedules, involvement in non-TB tasks, long hours at work, limited opportunities for vacation, resource inadequacy, work-related stigma, lack of promotional opportunities, and pay disparity and delay.

**Conclusion:**

Addressing work design, work-family balance and working conditions may increase the motivation and retention of LGTBS and improve human resources for TB at the district level and performance of the TB control programme.

## Introduction

In Nigeria, the local government area is the basic management unit of the National Tuberculosis and Leprosy Control Programme (NTBLCP) [1, 2]. The local government tuberculosis supervisor (LGTBS) coordinates tuberculosis (TB) control activities and oversees all the health facilities providing TB treatment services in each local government area. By being the link between the NTBLCP and health facilities and communities, the LGTBS has crucial roles in TB control including programme management and service delivery. Recently, the responsibility and workload for LGTBS in Nigeria have been gradually increasing with the expansion of TB control interventions including TB and human immunodeficiency virus collaborative activities, public-private mix, community TB care and drug resistant TB [1]. Thus, LGTBS are the largest cadre of dedicated TB control health workforce in Nigeria [3].

A well performing health workforce, that is responsive, efficient and effective [4], is important for infectious disease control [5], Especially at the district level for implementation of disease control programs and confronting public health challenges [6]. Nevertheless, inadequate health workforce is still a major impediment to disease-specific control programmes in low- and middle-income countries, where infectious diseases cause significant mortality, morbidity, and economic burden [5]. TB control in low-resource countries suffer from low numbers of healthcare workers, imbalances in staff distribution, poor quality of existing workforce, low human resource production, effects of human immunodeficiency virus infection, and poor motivation and retention [7–9].

Motivation, described as an individual’s willingness to exert and maintain efforts towards organisational goals, results from interaction between individuals and their work environment, and the fit between these interactions and broader societal context [10]. Conversely, retention entails preventing people from leaving an organisation to work elsewhere by giving due attention to the work environment [11]. Intention to leave, defined as individual’s own estimated probability of leaving one’s current job within the near future [12], is an important predictor of retention [13]. Job satisfaction, described as the affective orientation that employees have toward their work [14], mediates the influence of motivation on retention of health workers [15]. Thus, retention is a behavioural consequence of the level of motivation and job satisfaction [15], resulting from satisfaction with working conditions and job characteristics including work autonomy, clarity of roles, workload management, flexibility, work-life balance, child care, and support for career and professional development which improve their wellbeing and performance [16, 17].

Improving employee wellbeing and organisational performance are the focus of quality of work life (QWL), defined as the employee’s perceptions of how the working conditions in an organization can satisfy their important personal needs and work needs while achieving the organization’s goals [18]. QWL encompasses feelings about job content, physical work environment, pay, benefits, promotions, autonomy, teamwork, participation in decision-making, occupational health and safety, job security, communication, colleagues and managers support and work-life balance [19]. Organizations with high level of QWL record high productivity; low turnover and high job satisfaction [18, 20]. Organizations that intend to achieve high QWL must adopt a socially responsible approach to looking after their employees and create a good working environment as part of the total reward system [21].

Studies examining QWL among health workers found low overall QWL [18, 22–24]; moderately high QWL [20, 25–27] and high QWL [28, 29]. The findings of relationship of socio-demographics with QWL varied with contexts in different studies [18, 22, 24, 25, 30]. Quality of work life positively correlated with motivation [22, 29], which in turn, highly correlated with intention to leave [14, 22, 31]. Although QWL negatively correlated with intention to leave [22, 32–34], QWL influenced intention to leave indirectly through burnout [33]. Yet, despite the high job demand and increasing complexity of TB control interventions, we found no study that assessed the relationship between QWL, motivation and intention to leave of healthcare workers involved in TB control programme. Research on TB control workforce has been limited to resource availability and basic working conditions [3, 5, 8, 9, 35–38]. For instance, lack of staff and insufficient trained staff limited TB control and care and increased workload of existing staff in many settings [35–38]. In 2006, Nigeria’s TB control programme fell short of the optimum population-weighted density of 0.5 TB control supervisor per 1000 population resulting in a shortfall of at least 200 TB supervisors [3]. Evidence of employee-centred human resource practices that address tasks and roles, communication and motivation, although crucial to wellbeing of LGTBLS and improved TB control, is lacking. Thus, the NTBLCP must pay attention to the job characteristics and working conditions of local government (district) supervisors.

The purpose of this study was to examine the role of QWL in motivating and retaining LGTBS in Nigeria. This information would be useful for evidence-informed human resources planning for TB control at the district levels. District TB officers working in similar contexts and health decision makers could use this evidence to develop and implement quality improvement strategies to create a good working environment for improving the QWL of district TB officers in resource-poor countries with a similar TB prevalence as that in Nigeria.

## Materials and methods

### Study setting

The south-east region in Nigeria comprises 5 states namely Abia, Anambra, Ebonyi, Enugu and Anambra states delineated into 95 Local Government Areas (districts). The people are predominantly Igbo, Christians and rural dwellers. There are few minority ethnic groups like Igala. Within the region, sputum microscopy and TB treatment are provided free of charge in the publicly owned health facilities. Private health facilities also collaborate with the national TB control programme.

### Research design

The study adopted a mixed methods design. The quantitative strand involved cross-sectional questionnaire survey and the qualitative component, focus group discussion (FGD).

### Sampling and sample size

#### Quantitative

The respondents were all working as LGTBS in the region. We did not need to calculate sample size in this study since all LGTBS in the region were eligible for inclusion and given equal opportunity to participate in the study. However, 92 of 95 LGTBS responded by filling and returning the questionnaire. Eighty-seven (87) complete questionnaires were appropriate for analysis, resulting in about 92% net response rate. Three LGTBS were unavailable during the data collection period.

#### Qualitative

We purposively selected 26 focus group participants from among survey respondents. The LGTBS must have worked in the NTP for at least one year and willing to participate in the FGD. Participants were recruited from LGTBS attending workshops in Enugu, Nigeria. To ensure maximum variation, each group included both male and female gender participants from different States with varying level of education and tenure.

### Data collection tool and data collection

#### Quantitative

A pre-tested, self-administered questionnaire was used to collect data on the socio-demographic characteristics and QWL of LGTBS during their quarterly review meetings in 2016. The questionnaire was divided into four sections. Section A: sought information on state, gender, age, marital status, education and tenure in the TB control programme. Section B: was on quality of work life. To measure QWL and its dimension (work design, work context, work-family balance and work relevance), this study adapted questions from validated questionnaires used in previous studies [29, 39, 40]. Section C consisted of three questions that assessed the motivation of LGTBS sourced from an earlier study [29]. Section D also comprised three questions that assessed the intention to leave of LGTBS sourced from a previous study [29]. The draft questionnaire consisted of 54 QWL items and 3 items on motivation scored on a 7-point Likert scale (strongly disagree = 1, disagree = 2, somewhat disagree = 3, undecided = 4, somewhat agree = 5, agree = 6, strongly agree = 7).

The data collection tool (S1 Appendix), which was designed in English, was validated for content by two health management experts, one from the University of Nigeria Enugu Campus and the other, from the NTBLCP. The comments of the experts were helpful in rephrasing some questions. The questionnaire was also pre-tested on 10 LGTBS working in south-south Nigerian states to check for clarity and its completion time. The questionnaire was easy to understand and took about 30 minutes to complete.

#### Qualitative

We conducted three (3) focus group discussions (FGDs) with 26 TB supervisors to gain deep understanding of the quality of worklife factors influencing their motivation. Each focus group comprised 8 to 10. The FGDs were held at a venue and a time chosen in consultation with the participants. FGDs were conducted using discussion guide developed based on the dimensions of quality of worklife (S2 Appendix). Discussions, which lasted about 90 minutes, were held in English language and audiotaped with participants’ consent. Data collection was stopped after the third FGD when data saturation was reached and no new information emerged [41].

### Data analysis

#### Quantitative

Data from the questionnaires were entered, cleaned and analysed using SPSS version 20. Exploratory factor analysis (EFA) using principal component analysis (PCA) with varimax rotation was used to examine the similarity of the factor structure between the QWL scale and data collected [42]. The suitability of data for factor analysis was checked using Kaiser-Meyer-Olkin (KMO) measure of sampling adequacy and Bartlett’s test of sphericity. Since KMO statistics ranges between 0 and 1 and values greater than 0.5 are considered adequate, a KMO value of 0.6 was used as cut-off for sampling adequacy in this study [43]. The criterion for factor extraction was eigenvalues greater than 1.0. Communalities were used as the criterion for item deletion before rotation, whereas item loading was used as the standard for item deletion after rotation. Since communalities commonly ranged from 0.4 to 0.7 and item loading ≥0.5 represents adequate item loading [42], a value of 0.5 was used as the cut-off for item deletion before and after rotation in this study [29]. Cronbach’s alpha was used to assess the reliability of the extracted factors for each dimension and the overall QWL scale.

Univariate analyses of quality of work life (QWL), motivation and intention to leave were done using descriptive statistics (mean, standard deviation, frequencies and percentages). Bivariate analysis of association of quality work life, motivation and intention to leave with socio-demographic characteristics of LGTBS was done using non-parametric tests (Mann-Whitney test and Kruskal-Wallis test). Correlation between variables were done using Spearman correlation coefficient. We used multivariable linear regression to establish the predictors of QWL, motivation and retention of LGTBS. Statistical significance was set at alpha 0.05 level.

#### Qualitative

A thematic framework approach involving coding, mapping and organising the data under themes and interpretation was used to analyse the data [44]. Verbatim transcription of FGDs were prepared and imported into NVivo 11 software. Two persons coded the data using a codebook. The main themes were deduced from the dimensions of quality of worklife. The sub-themes were generated, inductively, by reading the transcripts and reflected job characteristics affecting motivation of TB supervisors. Inter-coder differences were resolved by consensus. The findings were validated during TB supervisors’ quarterly review meetings. We used excerpts and illustrative quotes to ground our findings in the data.

#### Triangulation

We analysed each data set from the quantitative and qualitative components separately and triangulated the findings at interpretive level to offset the weakness of each data collection method and enrich the findings from both sources [45].

### Ethical consideration

Ethical clearance was obtained from the Health Research Ethics Committee of the Ministry of Health, Enugu State Government, Nigeria. Written, informed consent was obtained from each participant. Completed questionnaires and audiotapes were kept confidential and stored in a safe place.

## Results

### Quantitative findings

The validated QWL scale consisted of 40 items (with 11 –work design, 19 –work context, 2 –work-family balance, and 8 –work relevance). The Kaiser-Meyer-Olkin (KMO) measure of sampling adequacy was 0.608 (X2 = 1936.132, ρ = 0.000). Fourteen items were deleted from the initial QWL questionnaire; 9 for low communalities and 5 because of inadequate loading (Table 1). Overall, the reliability of the resultant 40-item QWL questionnaire was 0.875. The reliability coefficients of the work design, work context, work-family balance, and work relevance dimensions were 0.774, 0.812, 0.673, and 0.724 correspondingly.

**Table 1:** Items deleted during analysis for different reasons, 2016.

Item	Reason for deletion
WD9 There are enough of health workers in the program to do the job properly	Inadequate item loading
WFB1 I am able to easily balance work and family life	Low communalities
WFB2 It is easy to leave during the work day to attend to dependent relations	Low communalities
WFB5 In this program, I am able to take my annual leave when I want	Low communalities
WFB6 I do not have to make changes to my plan for family or personal activities due to program related duties	Low communalities
WC6 I have a chance to suggest improvement to my control officer	Low communalities
WC22 The TB program offers study leave with pay for further professional education	Low communalities
WC1 I communicate well with my Control Officer	Inadequate item loading
WC14 I communicate well with my colleagues (other TB supervisors)	Inadequate item loading
WC23 It is important for TB supervisors to have a chance to attend regular continuing education programs	Inadequate item loading
WR1 I make significant contribution to service quality in TB control program	Low communalities
WR4 The general public has correct image of TB program staff	Low communalities
WR11 Other vertical programs offer better incentives	Low communalities
WR8 It is important to provide LGTB supervisors financial incentives	Inadequate item loading

Eighty-seven LGTBS returned valid questionnaires appropriate for analysis, indicating 92% net response rate. Most respondents were age ≥40 years (mean age = 46.55 years), married and have worked in TB programme for an average period of about 15 years. About 60% of the LGTBS possess at least a bachelors’ degree (Table 2).

**Table 2:** Mean scores (SD)^1^ of overall quality of work life, dimensions of quality of work life, motivation and intention to leave by demographics.

SDF		QWL		Work-family bal.		Work design		Work context		Work relevance		Motivation		Retention	
	n (%)	Mean	SD	Mean	SD	Mean	SD	Mean	SD	Mean	SD	Mean	SD	Mean	SD
Gender^**2**^															
Male	45 (52)	5.18	0.86	5.02	1.63	4.67	1.13	5.76	0.65	4.71	1.06	5.76	1.21	2.60	1.76
Female	42 (48)	5.12	0.92	4.59	1.84	4.57	1.11	5.81	0.63	4.83	1.08	6.10	0.82	2.76	1.39
Age^**2**^															
<40years	12 (14)	5.25	1.06	4.83	1.70	4.83	1.34	5.75	0.75	4.67	1.15	6.00	0.85	2.75	1.36
≥40years	75 (86)	5.13	0.86	4.81	1.75	4.59	1.08	5.79	0.62	4.79	1.07	5.91	1.08	2.67	1.63
Marital status^**2**^															
Married	79 (91)	5.13	0.90	4.77	1.75	4.59	1.12	5.77	0.66	4.75	1.07	5.85^*^	1.05	2.7	1.64
Not Married	8 (9)	5.38	0.74	5.25	1.67	4.88	1.13	5.89	0.35	5.00	1.20	6.63	0.74	2.50	0.93
Education^**2**^															
<Bachelors	35 (40)	5.49^*^	0.70	5.37^*^	1.57	5.03^*^	0.86	5.91	0.56	5.00	1.06	6.11	0.83	2.43	1.42
≥Bachelors	52 (60)	4.92	0.93	4.44	1.75	4.35	1.19	5.69	0.67	4.62	1.07	5.79	1.16	2.85	1.68
Tenure^**3**^															
<10years	25 (29)	5.16	0.94	4.76	1.61	4.56	1.23	5.92	0.70	4.68	1.07	5.84	1.31	3.04	1.70
10-19years	39 (45)	5.23	0.96	5.03	1.94	4.67	1.20	5.82	0.56	4.97	1.06	5.92	0.98	2.69	1.64
≥20years	23 (26)	5.00	0.88	4.52	1.50	4.61	0.84	5.57	0.66	4.52	1.08	6.00	0.85	2.26	1.32
State^**3**^															
Abia	17(19)	5.41^*^	0.71	5.47^*^	1.18	4.94^*^	0.90	5.59	0.94	4.88^*^	1.05	6.06^*^	0.75	2.76^*^	1.52
Anambra	20 (23)	4.90	0.72	4.40	1.64	4.35	0.99	5.85	0.49	4.45	1.00	5.85	0.93	2.40	1.35
Ebonyi	13 (15)	5.85	0.69	5.69	1.18	5.54	0.88	6.08	0.28	5.31	1.03	6.69	0.48	1.31	0.63
Enugu	17 (19)	4.53	1.01	3.82	1.94	3.65	1.17	5.82	0.53	4.17	0.95	5.06	1.43	4.18	1.85
Imo	20 (23)	5.25	0.79	4.95	1.93	4.85	0.81	5.65	0.67	5.15	1.04	6.10	0.79	2.50	1.05

^1^Reported on 1–7 scale with higher values.

^2^Mann-Whitney test.

^3^Kruskal-Wallis test.

*Significant at p value < 0.05

The mean scores (standard deviation) of overall QWL, work design, work-family life balance, work context and work relevance were 5.15 (0.88), 4.82 (1.74), 4.62 (1.11), 5.78 (0.64) and 4.77 (1.08) respectively. The mean score of motivation and intention to leave were 5.92 (1.05) and 2.68 (1.59) correspondingly. In the bivariate analysis (Table 2), motivation differed significantly by marital status. Overall QWL, work-family balance and work design differed significantly by education. Overall QWL, work-family balance, work design, work relevance, motivation and intention to leave differed significantly across states.

Quality of work life and its dimensions are positively inter-related (Table 3). Education was negatively associated with work-family balance (r = -0.271, ρ < 0.05), work design (r = -0.286, ρ < 0.01) and overall quality of work life (r = -0.317, ρ < 0.01). Tenure was negatively correlated with gender (r = -0.251, ρ < 0.05), but positively correlated age (r = 0.439, ρ < 0.01) and state (r = 0.234, ρ < 0.05). Motivation was positively correlated with marital status (r = 0.251, ρ < 0.05), work design (r = 0.571, ρ < 0.01), work relevance (r = -0.471, ρ < 0.01), overall quality of work life (r = -0.471, ρ < 0.01), but negatively correlated with intention to leave (r = -0.365, ρ < 0.01). Intention to leave was also negatively associated with work design (r = -0.266, ρ < 0.05), work context (r = -0.214, ρ < 0.05) and overall quality of work life (r = -0.242, ρ < 0.05).

**Table 3:** Correlation between variables.

		1	2	3	4	5	6	7	8	9	10	11	12	13
1	Gender	1.000												
2	Age	-0.147	1.000											
3	Marital status	0.170	-0.103	1.000										
4	Education	-0.052	0.012	-0.063	1.000									
5	Tenure	-0.251*	0.439**	-0.044	0.007	1.000								
6	State	0.074	0.199	-0.034	0.083	0.234*	1.000							
7	Work-famiy balance	-0.116	-0.001	0.080	-0.271*	-0.057	-0.087	1.000						
8	Work design	-0.046	-0.102	0.077	-0.286**	-0.021	-0.076	0.560**	1.000					
9	Work context	0.052	0.005	0.042	-0.182	-0.206	-0.028	0.224*	0.196	1.000				
10	Work relevance	0.074	0.034	0.076	-0.177	-0.047	0.072	0.365**	0.592**	0.157	1.000			
11	Overall quality of worklife	-0.027	-0.060	0.093	-0.317**	-0.084	-0.075	0.794**	0.807**	0.389**	0.671**	1.000		
12	Motivation	0.120	-0.008	0.251*	-0.117	-0.004	-0.033	0.182	0.571**	0.178	0.471**	0.471**	1.000	
13	Retention	0.108	-0.049	0.002	0.116	-0.174	0.095	-0.052	-0.266*	-0.214*	-0.136	-0.242*	-0.365**	1.000

*Correlation is significant at 0.05 level (2-tailed)

**Correlation is significant at 0.01 level (2-tailed).

Education predicted overall quality of work life (β = -0.554, ρ value < 0.05), work-family balance (β = -0.919, ρ value < 0.05) and work design (β = -0.670, ρ value < 0.05); whereas tenure predicted work context (β = -0.242, ρ value < 0.05) as in Table 4. Motivation of LGTBLS was predicted by work-family balance (β = -0.199, ρ value < 0.05) and work design (β = 0.514, ρ value < 0.05), which accounted for about 38% variance in motivation (Table 5). However, overall quality of work life and dimensions of quality of work life did not predict intention to leave. Motivation predicted intention to leave (β = -0.704, ρ value < 0.05) and accounted for about 29% variance in intention to leave (ρ < 0.05).

**Table 4:** Socio-demographic predictors of quality of worklife and its dimensions.

Demographics	Overall QWL		Work-family balance		Work design		Work context		Work relevance	
	B	Sig.	B	Sig.	B	Sig.	B	Sig.	B	Sig.
(Constant)	6.278	0.00	6.853	0.00	6.166	0.00	6.030	0.00	4.738	0.00
Gender	-0.133	0.50	-0.563	0.15	-0.139	0.58	-0.037	0.79	0.043	0.86
Age	-0.023	0.94	0.167	0.78	-0.294	0.44	0.245	0.28	0.232	0.54
Marital status	0.212	0.52	0.530	0.41	0.215	0.60	0.097	0.68	0.222	0.59
Education	-0.554	0.01*	-0.919	0.02*	-0.670	0.01*	-0.227	0.11	-0.389	0.11
Tenure	-0.077	0.59	-0.192	0.50	0.087	0.63	-0.242	0.02*	-0.132	0.47
State	-0.026	0.69	-0.080	0.54	-0.042	0.62	0.028	0.57	0.057	0.49
R Square	0.114		0.108		0.110		0.093		0.049	

*Significant p value < 0.05

**Table 5:** Predictors of motivation and intention to leave of local government TB supervisors.

	Motivation		Retention^1^		Retention^2^	
	B	P-value	B	P-value	B	P-value
(Constant)	2.241	0.015	6.600	0.000	8.178	0.000
Overall QWL	0.180	0.600	-0.840	0.171	-0.713	0.209
Work-family balance	-0.199	0.048*	0.305	0.086	0.165	0.324
Work design	0.514	0.003*	-0.342	0.249	0.020	0.946
Work context	0.143	0.392	-0.141	0.633	-0.040	0.883
Work relevance	0.107	0.398	0.278	0.216	0.354	0.091
Motivation					-0.704	0.000*
R Square	0.384		0.157		0.290	
						

*Significant p value < 0.05

### Qualitative findings

Table 6 shows the socio-demographic characteristics of FGD participants. The findings from the FGDs are presented thematically using the dimensions of quality of worklife namely work design, work context, work-family balance and work relevance.

**Table 6:** Socio-demographic characteristics of FGD participants (n = 26).

Variables	Categories n (%)	
Gender*	Male	16 (61.5)
	Female	10 (37.5)
Age	<40years	4 (15.4)
	≥40years	22 (84.6)
Marital status	Married	24 (91.3)
	Not Married	2 (7.7)
Education*	<Bachelors	11 (42.3)
	≥Bachelors	15 (57.7)
Tenure*	<10years	7 (26.9)
	10-19years	13 (50.0)
	≥20years	6 (23.1)
State*	Abia	6 (23.1)
	Anambra	5 (19.2)
	Ebonyi	4 (15.4)
	Enugu	7 (26.9)
	Imo	4 (15.4)

*Criteria for selecting FGD participants to ensure maximum variation

#### Work design

Autonomy and control at work, and workload emerged as key themes related to work design. LGTBLS stated that they have opportunity to use and develop human capacities on their job but noted that staff attendance monitoring policy of “*clocking in and clocking out*” (P7, FGD3) at the Local Government health department interrupted their daily work schedules. Some LGTBS are involved in other health programmes, which interfere with their TB work and increased their workload. Yet, some LGTBS relied on NTBLCP policy that “*on no condition will TB supervisor be assigned any other work apart from TB”* (P8, FGD1) to get exemption from non-TB tasks. Despite expansion of the TB control programme, most TB supervisors lack programme management team. Furthermore, most LGTBS are also TB service providers.

#### Work family balance

TB supervisors noted that high workload and long working hours deprive them time to attend to family needs and leave them exhausted after work. The NTBLCP seemed to have a culture of sending impromptu invitations for trainings and meetings, which “leaves LGTBS little time to meet family obligations before leaving for such meetings” (P5, FGD1). It was particularly more challenging for female supervisors to combine family needs and demanding TB programme responsibilities. Yet, LGTBS work for several years without vacation. As observed by one LGTBS, *“the work is demanding in the sense that I have been in the programme for twelve years*. *Could you imagine*, *for twelve years*, *I have not gone on leave”* (P1, FGD2).

#### Work context

Safe and healthy working environment, resource availability, career development and growth, and social integration were key themes related to the work context.

LGTBS stated that most TB treatment centres have inadequate facility design to support TB infection control, which exposes people to the hazard of contracting TB. Infection control measures have been limited to training of some TB staff, cough etiquette and respiratory hygiene and natural ventilation. As pointed by one LGTBS, *“we need standard operating procedure [for TB infection control] because one of our TB supervisors died because of TB of the bone after retirement and another TB service provider right now is having TB of the bone”* (P7, FGD2).

LGTBS stated that TB supervisors do not receive managerial support and allowances from the local government and management of some hospitals. *“They (policymakers) don’t regard health workers*. *We are on our own*. *… Yes*, *if we go*, *they will tell us that we should go and meet the NGOs that are supporting the programme”* (P6, FGD3*)*. LGTBS agreed that *“government need to be actively involved rather than leaving the funding to the partners*. *What if the partners are not with us again*?*”* (P4, FGD2). LGTBS mentioned that lack of functional motorcycles hinders supervision of TB program. LGTBS described the experience as traumatizing, *“when your motorbike gets old*, *but you can’t get a replacement”* (P3, FGD3). In 3 states, the ban on use of motorcycles within the capital city limits TB control activities. *Despite the waiver on publicly owned motorcycles*, *the enforcement officers still impounded LGTBS’s motorcycles* (P6, FGD1). LGTBS stressed the need for government to provide them means of transport or funding for supervision.

LGTBS mentioned that irregular and interrupted drug supply adversely affected quality of TB services. It was found that a logistics partner, contracted to distribute TB drugs, scarcely involved LGTBS, drug distribution is often delayed, and drugs meant for an entire local government are sent to one health facility. Yet, *when there is issue in these health centers*, *maybe*, *stock-out*, *we are the one that will bear the brunt* (P1, FGD2). LGTBS argued that the *“logistics partner receive adequate funding for the distribution of drugs; and if funds are equally made available to supervisors*, *they will do exactly what the partner were meant to do*, *and do it better*” (P3, FGD1).

LGTBS stated that delayed promotion, embargo on government-sponsored in-service trainings and trainings that are not linked to conversion and promotion limited their career development and growth. A LGTBS remarked, *“We have been to Zaria (National TB Training School)*, *but it is not recognized*. *They cannot use that certificate to promote you”* (P3, FGD2). Even when LGTBS obtained study leave without pay or chose part-time studies, at the completion of the training, conversion may be granted but the candidate’s grade level may be stepped down.

LGTBS stated that social interaction between TB supervisors and other staff of the Local Government is poor due to stigma associated with TB. A LGTBS observed, “*Once my colleagues see me*, *they just call me*, *‘TB*!*’ My name has been changed to TB”* (P5, FGD1). The TB supervisors explained that stigma associated with TB resulted in poor attention to TB control programme by decision makers, stigmatization of LGTBS by co-workers and refusal of health workers to be posted to TB programme.

#### Work relevance

Adequate and fair compensation, and job significance and recognition emerged as key themes related to work relevance. Most TB supervisors agreed that their compensation “*cannot be quantified with the work we do*” (P8, FGD3) due to disparity in salary scale, delay in payment, under-payments (salary cuts), and outright non-payment of staff salaries. As one LGTBLS observed, “*one can’t go home every day feeling happy*, *especially when one compares one’s salary with what those of one’s colleagues in the ministry receiving consolidated health salary take home”* (P3, FGD1). LGTBS held the view that “*workers are even neglected as the disease condition itself”* (P1, FGD3). Nonetheless, positive clinical outcomes for their patients give LGTBS sense of accomplishment and improve their quality of worklife. *“Even though the government does not pay us adequately*,*… we feel happy and fulfilled whenever we see our patients recovering*” (P9, FGD1).

## Discussion

The study revealed that the final QWL scale with 40 items was loaded on four dimensions. The extracted components were labelled work design, work-family life balance, work context and work relevance. Content-related validity was assured by the process of item generation which involved adaptation from literature and review of tool by experts to improve the content coverage and relevance to local context. Construct validity of the QWL scale was confirmed by adequate factor loading of the four components, which exceeded 0.5 cut-off [29, 42]. The QWL scale and its four dimensions also showed a good internal consistency with observed reliabilities within the acceptable range. Therefore, the 40-item QWL scale was found to be a valid and reliable measure of the QWL of LG TB supervisors in this study.

The overall quality of work life among TB supervisors in south-east Nigeria was found to be high which is consistent with results from previous studies [28, 29], but contrasts low overall QWL found in other settings [18, 22–24]. The dimensions of QWL varied from moderate (work-family life balance, work design and work relevance) to high QWL (work context), which is similar to the findings from other studies [20, 25–27]. The finding that work design had the lowest mean score may be due to high workload, lack of program team, lack of opportunities for flexible working schemes, participation in non-TB services and involvement in frontline TB service delivery by most TB supervisors. With increasing complexity of TB control interventions, it is imperative to improve autonomy and control at work, clarify roles of LGTBS, encourage LGTBS to build and work with program teams, and ensure compliance with the policy excluding TB supervisors from non-TB tasks.

The study revealed that education inversely and significantly predicted satisfaction with overall QWL, work-family balance and work design which is similar to findings among hospital employees in Iran [40]. Although gender was not predictive of quality of worklife, we expected the contrary given that evidence from focus groups indicated that female supervisors seemed to have more challenges than their male colleagues balancing family obligations and increasing demand at work. It might be that with more education, TB supervisors increasingly demand autonomy and control of their work and flexible work schedules that bridge organisational and employee interests and balance work and family life.

The study finding that tenure inversely and significantly predicted satisfaction with the work context is consistent with results from a previous study [46], but differs from evidence elsewhere [24, 27]. Four factors from the qualitative sub-study could explain this finding. As the length of stay in TB control programme increases, LGTBS show higher concern for safe and health working environment especially concern for TB infection control; accumulate experiences with weak political commitment and resource inadequacy; detest negative organisational climate and stigmatising work environment; and worry about frustrating promotional opportunities. Job enrichment strategies focusing on safe and healthy environment, resource availability, social integration, management and leadership, and opportunities for career growth might improve the work context of LGTBS.

Even though no socio-demographic factors significantly influenced work relevance, its inverse relationship with education and tenure suggests that with more education and longer tenure, perception of work relevance diminishes among LGTBS. Insights from the FGDs reveal that despite not being adequately remunerated, a sense of accomplishment improves work relevance of LGTBS. Additionally, LGTBS perceive their job as not well unrecognised by government and the public. To improve work relevance of LGTBS, it is imperative that LGTBS receive adequate and fair pay and their contributions to the NTP and society at large acknowledged.

The motivation of LGTBS in south-east Nigeria was found to be high, which is consistent with result from a previous study [14]. Furthermore, the study revealed that work design and work-family balance predicted motivation among LGTBS, which is consistent with existing evidence [16, 17, 22, 33]. In this study, LGTBS were satisfied with factors arising from intrinsic conditions of their job namely autonomy and control at work, responsibility, learning opportunities in TB control programme, achievement of positive clinical outcomes for their patients, and acknowledgement and recognition from cured patients. Whereas extrinsic conditions such as the supervisory practices and support from health development partners and NTBLCP enhanced their motivation, LGTBS were dissatisfied with organisational policies including limited opportunities for vacation, involvement in non-TB tasks, long hours at work, lack of flexible work schedules, impromptu invitation to workshops and non-replacement of motorcycles. Improving motivation of LGTBS must address these extrinsic factors, which seem to limit the work design and disrupt TB supervisors’ work-family balance.

We found that motivation mediated the relationship between quality of worklife and intention to leave and accounted for about 29% variance in intention to leave among LGTBS. Boma and Laschinger also found that whereas QWL correlated negatively with intention to leave, QWL influenced intention to leave indirectly through burnout [33]. In this study, over 70% of the LGTBS have been tenured for over ten years in the NTBLCP. The high level of motivation, which reflects TB supervisors’ satisfaction with the total work environment may explain their low intentions to leave the NTBLCP. However, efforts to improve motivation and retention of LGTBS must pay attention to the dimensions of quality of worklife, which focus on organisational requirements to achieve employee wellbeing, since needs satisfaction resulting from workplace experiences contribute to job satisfaction [47].

Our study was based on context-specific, valid and reliable QWL scale and provides baseline values for QWL scores and its relationship with motivation and intention to leave among LGTBS in Nigeria. Notwithstanding that the questionnaire survey provides only a snapshot of TB supervisors’ perceptions at the time of the study, the qualitative insights into the intrinsic and extrinsic conditions that motivate LGTBS enriched the findings. Also, generalisation of results may be limited by the scope of the study as only one of the six geopolitical zones in Nigeria was involved. Future studies in other geopolitical zones are needed to compare findings across settings. Equally, LGTBS may have provided some socially desirable answers to some questions in the questionnaire but triangulation of the quantitative and qualitative findings increased the validity of the study.

## Conclusions

This study has examined the levels of QWL and how QWL influences motivation and intention to leave among local government TB supervisors in south-eastern region of Nigeria. The findings reveal that LGTBS have high QWL and motivation, but low intention to leave their jobs. Whereas education predicted satisfaction with overall QWL, work-family balance and work design; tenure (years of work experience) predicted satisfaction with work context. Our study established that motivation mediated the relationship between QWL and intention to leave. Motivation was predicted by work design and work-family balance. So, to increase the motivation and retention of LGTBS, health decision makers should collaborate with LGTBS to improve TB supervisors’ workload management, work stress, work autonomy, flexibility, work-family balance and working conditions.

## Supporting information

S1 Appendix Questionnaire. (DOCX)

S2 Appendix Focus group discussion (FGD) guide. (DOCX)
