# Supplementary material for: The influence of quality of work life on motivation and retention of local government tuberculosis control programme supervisors in South-eastern Nigeria
Source: PLoS One. 2019 Jul 24;14(7):e0220292. doi: 10.1371/journal.pone.0220292 (PMC6655736; doi:10.1371/journal.pone.0220292)
Supplement: S1 Appendix — (DOCX) [file pone.0220292.s001.docx]

**Questionnaire**

**A study of relationship of quality of work life, motivation and intention to leave among Local Government TBL Supervisors in Southeastern Nigeria**

**Description**

Quality of worklife refers to employees’ satisfaction with the working life. It covers employee feeling about work-family balance, work design, work context and work relevance. The purpose of the study is to provide evidence of the effect of quality of work life on motivation and turnover intention among Local Government TBL Supervisors in Southeastern Nigeria. Such evidence is required by policy makers to inform policies to improve performance NTBLCP.

**Participation**

Your participation in this research project is voluntary. Your decision to participate or not to participate will in no way impact upon your work. You will be asked to complete the attached questionnaire which consists of three sections; quality of work life, organizational outcomes and socio-demographic data. It can be completed in about 30mins.

**Expected benefits**

There are no direct benefits to you in participating in this research. No incentives are offered. However, the result will have scientific interest that may eventually have benefits for the human resources for health management in the NTBLCP.

**Risks**

There are no risks associated with your participation or non-participation in this research.

**Confidentiality**

All comments and responses are anonymous and will be treated confidentially. The names of individual persons are not required in any of the responses.

**Consent to participate**

Please complete the informed consent form as an indication of your voluntary acceptance to participate in this study.

**Questions/further information about the project**

Please contact the principal investigator, Daniel Ogbuabor, on 08038774436 or email: [ogbuabordc@yahoo.com](mailto:ogbuabordc@yahoo.com) to have any question answered or if you require further information about the project.

**QUESTIONNAIRE**

**SECTION A: SOCIO-DEMOGRAPHIC AND JOB-RELATED FACTORS**

| 1 | Male |  |
| --- | --- | --- |
| 2 | Female |  |

1. Gender:

| 20-24 | 25-29 | 30-34 | 35-39 | 40-44 | 45-49 | 50-54 | ≥55 |
| --- | --- | --- | --- | --- | --- | --- | --- |
|  |  |  |  |  |  |  |  |

1. Age

| 1 | Never Married |  |
| --- | --- | --- |
| 2 | Married |  |
| 3 | Divorced |  |
| 4 | Widowed |  |

1. Marital Status
2. Highest Educational Attainment _______________________
3. Length of Service in the Program____________________
4. State _______________________________________

| **SECTION B: QUALITY OF WORKLIFE (QWL)**  This set of questions asks about your quality of working life as TBL Supervisors. Please indicate your response by ticking the appropriate box which most accurately reflects the extent to which you agree or disagree with each statement. | | | | | | | | |
| --- | --- | --- | --- | --- | --- | --- | --- | --- |
| **S/N** | **QWL ITEMS** | **Strongly disagree** | **Disagree** | **Somewhat Disagree** | **Undecided** | **Somewhat Agree** | **Agree** | **Strongly Agree** |
|  | **Work design (WD)** |  |  |  |  |  |  |  |
| 1 | I have considerable freedom in doing my job | 1 | 2 | 3 | 4 | 5 | 6 | 7 |
| 2 | My job provides me the opportunity of flexible work hours | 1 | 2 | 3 | 4 | 5 | 6 | 7 |
| 3 | My Control Officer does not interfere in my job | 1 | 2 | 3 | 4 | 5 | 6 | 7 |
| 4 | I have enough time to do my job well | 1 | 2 | 3 | 4 | 5 | 6 | 7 |
| 5 | I do not perform many tasks outside my job description | 1 | 2 | 3 | 4 | 5 | 6 | 7 |
| 6 | I have chance to try my own methods of doing the job | 1 | 2 | 3 | 4 | 5 | 6 | 7 |
| 7 | My workload is not heavy | 1 | 2 | 3 | 4 | 5 | 6 | 7 |
| 8 | I receive enough resources to do my job properly | 1 | 2 | 3 | 4 | 5 | 6 | 7 |
| 9 | There are enough of health workers in the program to do the job properly | 1 | 2 | 3 | 4 | 5 | 6 | 7 |
| 10 | My working environment is secure | 1 | 2 | 3 | 4 | 5 | 6 | 7 |
| 11 | My job provides for steady employment without frequent transfers | 1 | 2 | 3 | 4 | 5 | 6 | 7 |
| 12 | LG TBL Supervisors are treated fairly and ethically | 1 | 2 | 3 | 4 | 5 | 6 | 7 |
|  | **Work context (WC)** |  |  |  |  |  |  |  |
| 1 | I communicate well with my Control Officer | 1 | 2 | 3 | 4 | 5 | 6 | 7 |
| 2 | I receive regular and supportive supervision from my Control Officer | 1 | 2 | 3 | 4 | 5 | 6 | 7 |
| 3 | My Control Officer provides me feedback frequently about how I perform | 1 | 2 | 3 | 4 | 5 | 6 | 7 |
| 4 | My co-workers give me feedback about my performance on the job | 1 | 2 | 3 | 4 | 5 | 6 | 7 |
| 5 | Feedbacks help me do my job properly | 1 | 2 | 3 | 4 | 5 | 6 | 7 |
| 6 | I have a chance to suggest improvements to my Control Officer | 1 | 2 | 3 | 4 | 5 | 6 | 7 |
| 7 | My duties are clearly defined | 1 | 2 | 3 | 4 | 5 | 6 | 7 |
| 8 | I understand how my job contributes to the program's mission | 1 | 2 | 3 | 4 | 5 | 6 | 7 |
| 9 | I know exactly what is expected of me on my job | 1 | 2 | 3 | 4 | 5 | 6 | 7 |
| 10 | I have the guidelines and information required to do my job | 1 | 2 | 3 | 4 | 5 | 6 | 7 |
| 11 | I am able to provide patient care according to standard guidelines | 1 | 2 | 3 | 4 | 5 | 6 | 7 |
| 12 | I feel confident that I am effective at getting my job done | 1 | 2 | 3 | 4 | 5 | 6 | 7 |
| 13 | I interact well with my colleagues (other LGTBL Supervisors) | 1 | 2 | 3 | 4 | 5 | 6 | 7 |
| 14 | I communicate well with other NTBLCP Program Staff | 1 | 2 | 3 | 4 | 5 | 6 | 7 |
| 15 | There is a team spirit towards NTBLCP’s vision in my State Program | 1 | 2 | 3 | 4 | 5 | 6 | 7 |
| 16 | Staff of other health programs (e.g. malaria, HIV/AIDS) respect me | 1 | 2 | 3 | 4 | 5 | 6 | 7 |
| 17 | I have cordial relationship with administrative staff in my LGA | 1 | 2 | 3 | 4 | 5 | 6 | 7 |
| 18 | I get along well with facility DOTS providers in my LGA | 1 | 2 | 3 | 4 | 5 | 6 | 7 |
| 19 | I am able to develop a wide variety of skills on my job | 1 | 2 | 3 | 4 | 5 | 6 | 7 |
| 20 | I have opportunity to fully utilize my skills and expertise | 1 | 2 | 3 | 4 | 5 | 6 | 7 |
| 21 | I have a fair chance to grow as a person and a TB Program Staff | 1 | 2 | 3 | 4 | 5 | 6 | 7 |
| 22 | The TBL Program offers study leave with pay for further professional education | 1 | 2 | 3 | 4 | 5 | 6 | 7 |
| 23 | It is important for TB Supervisors to have a chance to attend regular continuing education programs | 1 | 2 | 3 | 4 | 5 | 6 | 7 |
| 24 | I have a future in this program and will be able to take higher positions | 1 | 2 | 3 | 4 | 5 | 6 | 7 |
|  | **Work-family balance (WFB)** |  |  |  |  |  |  |  |
| 1 | I am able to easily balance work and family life | 1 | 2 | 3 | 4 | 5 | 6 | 7 |
| 2 | It is easy to leave during the work day to attend to dependent relations | 1 | 2 | 3 | 4 | 5 | 6 | 7 |
| 3 | My work schedule makes it easy for me to fulfill my family obligations | 1 | 2 | 3 | 4 | 5 | 6 | 7 |
| 4 | I am able to relax at home after my daily routine work | 1 | 2 | 3 | 4 | 5 | 6 | 7 |
| 5 | In this program, I am able to take my annual leave when I want | 1 | 2 | 3 | 4 | 5 | 6 | 7 |
| 6 | I do not have to make changes to my plan for family or personal activities due to program related duties | 1 | 2 | 3 | 4 | 5 | 6 | 7 |
|  | **Work relevance (WR)** |  |  |  |  |  |  |  |
| 1 | I make significant contribution to service quality in NTBLCP (Program) | 1 | 2 | 3 | 4 | 5 | 6 | 7 |
| 2 | The Program leadership recognizes staff accomplishments at work | 1 | 2 | 3 | 4 | 5 | 6 | 7 |
| 3 | The Program leadership has respect for LGTBL Supervisors | 1 | 2 | 3 | 4 | 5 | 6 | 7 |
| 4 | The general public has correct image of TBL Program Staff | 1 | 2 | 3 | 4 | 5 | 6 | 7 |
| 5 | My job significantly affects lives of individuals, families and communities | 1 | 2 | 3 | 4 | 5 | 6 | 7 |
| 6 | My job influences decisions that significantly affect the NTBLCP | 1 | 2 | 3 | 4 | 5 | 6 | 7 |
| 7 | I feel I am being paid a fair amount for the work that I do | 1 | 2 | 3 | 4 | 5 | 6 | 7 |
| 8 | It is important to provide LG TBL Supervisors financial incentives | 1 | 2 | 3 | 4 | 5 | 6 | 7 |
| 9 | I feel appreciated when I think about what they pay me | 1 | 2 | 3 | 4 | 5 | 6 | 7 |
| 10 | My pay is adequate for my job, given the current national pay schemes | 1 | 2 | 3 | 4 | 5 | 6 | 7 |
| 11 | Other vertical programmes offer better incentives | 1 | 2 | 3 | 4 | 5 | 6 | 7 |
| 12 | My salary meets my basic family needs | 1 | 2 | 3 | 4 | 5 | 6 | 7 |
| **SECTION C: MOTIVATION** | | | | | | | | |
| 1 | I am satisfied with my job | 1 | 2 | 3 | 4 | 5 | 6 | 7 |
| 2 | I am completely involved in my work | 1 | 2 | 3 | 4 | 5 | 6 | 7 |
| 3 | For me, this is the best of all possible programs to work for | 1 | 2 | 3 | 4 | 5 | 6 | 7 |
| **SECTION D: INTENTION TO LEAVE** | |  |  |  |  |  |  |  |
| 1 | It is likely that I will actively look for another job during the next year | 1 | 2 | 3 | 4 | 5 | 6 | 7 |
| 2 | I often think about quitting this program | 1 | 2 | 3 | 4 | 5 | 6 | 7 |
| 3 | If it were possible, I would like to get into a new program | 1 | 2 | 3 | 4 | 5 | 6 | 7 |

Thanks for participating in this research
