# Supplementary material for: The influence of quality of work life on motivation and retention of local government tuberculosis control programme supervisors in South-eastern Nigeria
Source: PLoS One. 2019 Jul 24;14(7):e0220292. doi: 10.1371/journal.pone.0220292 (PMC6655736; doi:10.1371/journal.pone.0220292)
Supplement: S2 Appendix — (DOCX) [file pone.0220292.s002.docx]

**Focus Group Discussion (FGD) Guide**

1. How satisfied are you with your job as TB supervisors?
2. Would you say that you have the autonomy and control to manage the programme in the local government area? Probe for flexible work schedules and staff attendance monitoring policy
3. How do non-programme tasks within your health department affect your role as TB supervisors?
4. Would you consider your work environment safe and healthy for you as TB supervisors? Probe for how infection control has affected TB supervisors’ work?
5. What is your relationship with decision makers? (chairmen of the local government, head of department of health and partners). Probe for technical and managerial support
6. How does resource availability or inadequacy affect your functioning as TB supervisors? Probe for motobikes; drug supply; recording and reporting material; electronic data transmission; human resources and funding.
7. Would you feel that you have opportunities for career development on this job as TB supervisors? Probe for – training, promotion, job security
8. How demanding is TB supervisors’ work?
9. How does your family lives affect your work as supervisors, and how does your work as supervisors affect your family lives?
10. How satisfied are you with your remuneration?
11. What are your experiences with how people in the community around you perceive the work you do?
12. What are your experiences of stigma at work as TB supervisors?
13. What is your most important / significant challenge in this work?
14. What should be done to improve the quality of work life of TB supervisors?
